# 

**Published:** 1895-08

**Authors:** 


					﻿WHEN WRITING TO ADVERTISERS, PLEASE SAY “ I SAW YOUR
AD. IN THE ATLANTA MEDICAL AND SURGICAL JOURNAL.”
CSTABUSHEO ItSS.	tUBSCRIPTION, ^,..0A
ATLANTA
IWEDIGKIi IlflD SUHGIGlfj
JOURNAL.
LUTHER B. GRANDY, M.D.,	M. B. HUTCHINS, M.D.,
MANAGING EUITOB.	BUSINESS MANAGES.
COLI.ABORATOR8 ;
H. V. M. MILLER M.D., LL.D., VIRGIL O. HARDON, M.D., FLOYD W. McRAE, M.D.,
DUNBAR ROY, M.D., and H. R. SLACK, M.D., Ph.M.
NKWorries:	Atlanta, Ga., August, 1895.	No. 6.
«NERVOUS
PROSTRATION
<^4RSEN^yRfl
CHAS. ROOME PARMELE CO.. 98 william ST.. N.Y.
Poblishbd BT THE FRANKLIN PRINTING AND PUBLISHING 00., Aixakta, Gx.
Entered at the Poet-Offioe at Atlanta, OtL,, as seoond-olaea n^ter.
				

